# Leveraging root trait identification for winter wheat yield prediction using machine learning

**DOI:** 10.3389/fpls.2026.1809461

**Published:** 2026-07-17

**Authors:** Fei Zhao, Farhan bin Mohamed, Guofang Wang, Jiancheng Zhang, Na Yang, Wuping Zhang

**Affiliations:** 1 Faculty of Computing, Universiti Teknologi Malaysia, Johor Bahru, Johor, Malaysia; 2 College of Resources and Environment, Shanxi Agricultural University, Taigu, China; 3 Institute of Cotton Research, Shanxi Agricultural University, Yuncheng, China; 4 Faculty of Software Technologies, Shanxi Agricultural University, Taigu, China

**Keywords:** machine learning, mid-layer root, root traits, winter wheat, yield prediction

## Abstract

**Introduction:**

Accurate crop yield prediction remains challenging because yield formation is influenced by complex interactions between plant traits and soil environments across multiple soil layers. Although root traits are widely recognized as important for crop resource acquisition and yield formation, their relative contributions across soil depths and their interannual stability have not been fully quantified. This limits their effective use in trait-based yield prediction. Therefore, identifying key root indicators with stable predictive relevance and developing physiologically interpretable feature frameworks are important for improving the analysis and prediction of crop yield variation.

**Methods:**

Based on multi-year field experiment data, winter wheat root traits across the 0–60 cm soil profile were analyzed using a layer-wise approach. To ensure consistency with the scale of grain yield observations, multi-point and multi-layer root measurements were aggregated into plot-year-level feature variables before modeling. A trait-prioritization and feature-organization strategy integrating correlation analysis, interannual stability evaluation, weighting sensitivity analysis, and model-based contribution analysis was used to identify representative root traits and construct a hierarchical root-trait feature framework. These analyses were used to support trait prioritization and feature organization, rather than as prediction-optimized trait preselection prior to cross-validation. Representative machine-learning models from different categories were then compared under a unified preprocessing and year-grouped cross-validation workflow to evaluate the predictive performance of different root-trait combinations.

**Results and discussion:**

The results showed clear soil-depth dependency in the relationship between root traits and winter wheat yield. Root traits in the 20–30 cm soil layer showed the most stable and informative associations with yield variation under the present experimental conditions. Root dry matter density in the 20–30 cm layer (RDMD at 20–30 cm) was identified as the most representative single predictive trait, showing relatively strong performance in correlation strength, interannual stability, and model-based contribution. Further analyses indicated that incorporating additional root volume- and surface-area-related traits from the 20–30 cm layer improved predictive performance, while deep root distribution indicators from 30–60 cm provided complementary information. Comparative modeling results showed that nonlinear ensemble models generally performed better than linear models in capturing multi-layer root-trait information, with AdaBoost achieving the best cross-validated performance under the profile distribution–enhanced feature tier, with a mean R^2^ of 0.74. SHAP and permutation importance analyses further indicated that the most influential predictors were mainly concentrated in the 20–30 cm soil layer, particularly RVD, RSAD, RDMD, root dry mass, and root volume. Overall, this study highlights the functional differentiation of root traits across soil layers and supports a trait-based, physiologically interpretable framework for analyzing winter wheat yield variation. The proposed framework should be regarded as a preliminary analytical approach under the present experimental conditions and may provide a methodological reference for future studies that further integrate root traits with environmental and management information.

## 1 Introduction

Crop yield formation results from the combined effects of genetic characteristics, environmental conditions, and management practices. As the primary organ for water and nutrient acquisition, the root system plays a critical role in regulating crop growth, development, and yield formation (Uga et al., 2013; NirmalAruban et al., 2025). Previous studies have shown that root traits not only directly affect the efficiency of soil resource uptake, but also indirectly constrain biomass accumulation and final yield through regulating above–belowground carbon allocation processes (Freschet et al., 2010; Roumet et al., 2016). Therefore, elucidating the quantitative relationship between root traits and crop yield is essential for understanding resource-use mechanisms and improving yield prediction accuracy in crop production studies.

In agroecosystems, the spatiotemporal instability of water and nutrient supply further amplifies the importance of root traits in yield formation (Blum, 2011; Tardieu and Tuberosa, 2010). Increasing evidence indicates that the spatial distribution of roots, particularly the configuration of root traits across different soil layers, strongly influences crop growth performance and yield stability under water-limited conditions (Wasson et al., 2012; White and Kirkegaard, 2010). Compared with surface soils that are highly affected by precipitation and evaporation, middle and deep soil layers generally provide a more stable moisture environment. Consequently, deeper root extension has been widely regarded as an effective strategy for enhancing drought resistance and improving resource-use efficiency in crops (Lynch, 2013; Ehdaie et al., 2012; Kell, 2011). Against this background, functional differentiation of root traits across soil layers has become a major focus in root ecology and crop physiology research. However, existing studies mostly focus on single traits, specific soil layers, or single years, and still lack a systematic assessment of the stability of root characteristics’ contribution to yield across multiple soil layers. Currently, a systematic multi-layer assessment of the stability of root characteristics’ contribution to yield is still lacking.

The recently proposed root economics spectrum theory provides a new conceptual framework for understanding trade-offs among root traits and their ecological functions. This theory suggests that root traits exhibit systematic trade-offs between resource acquisition efficiency and construction costs, and that different trait combinations reflect distinct resource acquisition strategies (Freschet et al., 2010; Roumet et al., 2016). In cropping systems, this framework offers valuable insights into how root traits influence yield formation by regulating resource uptake and carbon allocation (Lynch, 2019). However, most existing studies have focused on individual traits or specific soil layers, and systematic multi-layer evaluations of the stability of root trait contributions to yield are still lacking.

Studies on wheat and other cereal crops have demonstrated that traits such as RLD, root surface area, root volume, and root dry weight are closely associated with water and nutrient uptake capacity and can influence crop growth advantages and yield potential (Wasson et al., 2012; Watt et al., 2020). Nevertheless, conclusions across studies remain inconsistent, partly because many investigations are based on single-year experiments or specific environmental conditions. In addition, strong collinearity among root traits is common, which limits the stability and transferability of results in yield prediction applications (York et al., 2013; Zobel, 2011). Consequently, identifying representative root traits with high interannual stability from multidimensional root trait datasets remains a major challenge.

Although significant progress has been made in crop yield prediction, most studies rely on meteorological variables, remote sensing indicators, or aboveground traits (Lobell et al., 2015; Jeong et al., 2016). However, these approaches often fail to represent belowground processes governing resource acquisition. In fact, crop productivity largely depends on root-mediated water and nutrient uptake (Lynch, 2013; Wasson et al., 2012). Root systems function as the key interface between plants and soil, and their spatial distribution across soil layers strongly influences resource capture and crop performance (White and Kirkegaard, 2010; Lynch, 2019). Therefore, incorporating root traits into yield prediction models may provide physiologically meaningful predictors that explicitly represent belowground resource acquisition processes that are not captured by conventional yield prediction variables.

With advances in statistical analysis and machine learning techniques, multivariate modeling approaches have increasingly been applied to crop yield prediction to integrate multiple phenotypic traits and improve predictive performance (Shahhosseini et al., 2021; Van Eeuwijk et al., 2019). Compared with traditional univariate analyses, multivariate models are better suited to capturing nonlinear relationships and interaction effects among traits. However, model performance largely depends on the rational construction and selection of input features. Without physiologically meaningful and stable key traits, models are prone to overfitting or poor generalization (James et al., 2013). In root research, previous studies have pointed out that the direct application of root traits in yield prediction is still constrained by high data acquisition costs, trait redundancy, and insufficient interannual stability (Lynch, 2019). Therefore, it is crucial to develop a yield prediction method that combines physiological interpretation with data-driven modeling.

To better integrate physiologically meaningful predictors into yield prediction models, multivariate statistical and machine learning approaches have increasingly been applied, multivariate modeling approaches have increasingly been applied to crop yield prediction to integrate multiple phenotypic traits and improve predictive performance (Shahhosseini et al.,2021; van Eeuwijk et al., 2019). Compared with traditional univariate analyses, multivariate models are better suited to capturing nonlinear relationships and interaction effects among traits. However, model performance largely depends on the rational construction and selection of input features. Without physiologically meaningful and stable key traits, models are prone to overfitting or poor generalization (James et al., 2013). In root research, previous studies have pointed out that the direct application of root traits in yield prediction is still constrained by high data acquisition costs, trait redundancy, and insufficient interannual stability (Lynch, 2019).

Based on this background, this study focuses on winter wheat and systematically analyzes the relationships between root traits and yield within the 0–60 cm soil profile. Emphasis is placed on the consistency of root traits in terms of correlation strength, interannual stability, and contribution to multivariate predictive models. Furthermore, by comparing multiple machine learning models, this study evaluates the predictive performance of different root trait combinations and constructs a hierarchical root-trait feature framework tailored to different prediction objectives. The goal is to identify root traits that are critical for winter wheat yield prediction and to provide a preliminary trait-based methodological reference for understanding root–yield relationships under the experimental conditions of this study.

## 2 Materials and methods

### 2.1 Study area

**Figure 1:**
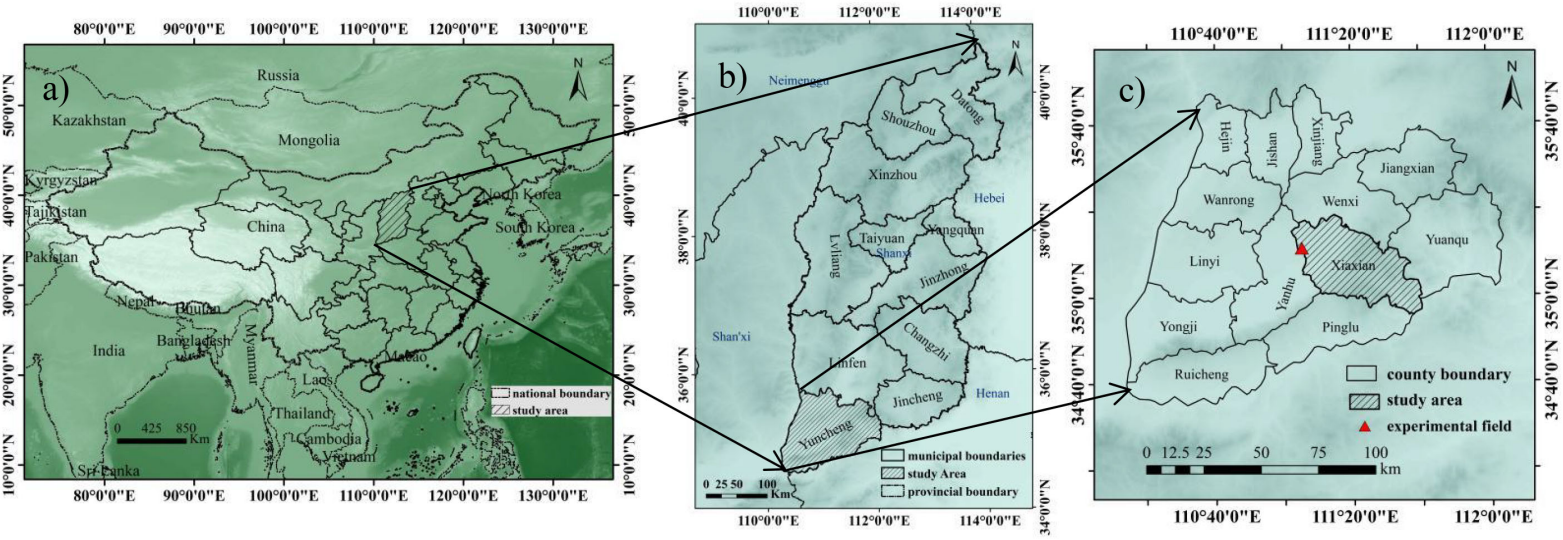
Location of the study site.

The experimental site was in Xia County, Yuncheng City, Shanxi Province, at the Shuitou Experimental Base of the Cotton Research Institute, Shanxi Agricultural University (35° 11′ 22″ N, 111° 05′ 17″ E) (**Figure 1**). The study area lies in the Fen–Wei Plain on the eastern Loess Plateau, at an elevation of 407.5 m. The region has a mean annual temperature of 13.3 °C, an average annual sunshine duration of 2039 h, a frost-free period of 212 days, and a mean annual precipitation of 525 mm. Precipitation is mainly concentrated in summer and autumn, with rainfall from June to September accounting for 64.2% of the annual total. The climate is classified as a temperate continental monsoon climate. The soil at the experimental site is classified as cinnamon soil, with a silty loam texture.

### 2.2 Experimental design

This study constructs a dataset based on a long-term field experiment on tillage and fertilization. This experiment, through long-term control of different agricultural management practices, provides an ideal platform for analyzing the spatiotemporal variability of root characteristics and their sustained impact on yield formation. The experiment was initiated in 2007 with winter wheat as the test crop. Wheat was sown in early October each year and harvested in early June of the following year, followed by a summer fallow period. No irrigation was applied during the wheat growing season, and a single cropping system was adopted. A two-factor randomized block design was employed, with four treatments (**Table 1**): conventional tillage without organic fertilizer application (T0M0), conventional tillage with organic fertilizer application (T0M1), no-tillage without organic fertilizer application (T1M0), and no-tillage with organic fertilizer application (T1M1). Each treatment was replicated three times, and each plot covered an area of 125 m^2^. After wheat harvest, straw was chopped and retained on the soil surface, with a stubble height of 15 comfort the conventional tillage treatments, plowing was conducted in mid-July to a depth of 25 cm. No-tillage plots received no soil disturbance prior to sowing. Organic fertilizer was applied before sowing in early October, while chemical fertilizers were applied simultaneously with mechanical sowing. The chemical fertilizers used were urea (46% N) and triple superphosphate (46% P_2_O_5_). The organic fertilizer was well-decomposed chicken manure, with an alkali-hydrolysable nitrogen of 18.6 mg·kg−1, P2O5 of 36.7 mg·kg−1, and K2O of 17.4 g·kg−1.All treatments received the same field management practices during the wheat growing season. Fertilizer application rates for each treatment are shown in **Table 1**.

**Table 1:** Fertilizer application rates for each treatment.

Treatment	Tillage	Organic fertilizer (kg hm^−2^)	N (kg hm^−2^)	P_2_O_5_ (kg hm^−2^)
T0M0	Conventional tillage	–	165	150
T0M1	Conventional tillage	15000	165	150
T1M0	No-tillage	–	165	150
T1M1	No-tillage	15000	165	150

### 2.3 Root sampling

Root traits of winter wheat were measured at key developmental stages, with root sampling conducted at the flowering stage from 2019 to 2022. Because the root system of winter wheat is relatively well-developed during the flowering period, and its role in water and nutrient absorption is closely related to reproductive growth and subsequent yield formation, root traits were measured during the flowering period. Therefore, we believe that under the current experimental conditions, the flowering period is a suitable observation window for assessing the relationship between root traits and yield. Root samples were collected using the excavation method. For each plot, three sampling points were selected. At each sampling point, samples were taken centered on a wheat row (row spacing of 20 cm), with a sampling area of 20 cm (perpendicular to the row direction) × 10 cm (along with the row direction) and a sampling depth of 60 cm, divided into layers at 10 cm intervals. Each treatment was replicated three times. Considering the four experimental years (2019–2022), the total number of root-layer samples used in the analysis was 864 (36 sampling points×6 layers×4 years).

The collected samples were placed into fine nylon mesh bags (0.1 mm mesh size), soaked in water, and then repeatedly washed under running water to remove adhering soil and impurities. After cleaning, the root samples were collected, sealed in zip-lock bags, and stored at −18 °C until further analysis.

### 2.4 Yield determination

Each year in early June, after wheat reached maturity, yield was determined by harvesting a sampling area consisting of five rows per plot, each 10 m in length. Grain yield was calculated and standardized to a moisture content of 13%.

### 2.5 Root trait measurement

The morphological traits of each group of fine roots were scanned via root analyzer system (WinRHIZO, Regent Instruments Company, Quebec, Canada) including total length, surface area, volume and average diameter. Based on these measurements, Root Length Density, Root Surface Area Density, Root Volume Density, and Root Dry Mass Density were calculated.

#### 2.5.1 Root length density (RLD)

Root Length Density(RLD) refers to the total length of roots per unit volume of soil and is a fundamental indicator describing the degree of root distribution within the soil. This parameter reflects the ability of roots to explore soil space and is closely related to crop water and nutrient uptake efficiency. Higher RLD values generally indicate a denser root distribution and greater potential for soil resource utilization (Böhm, 1979). The calculation is shown in Equation (1):


$$\text{RLD}=\frac{\text{L}}{{\text{V}}_{\text{s}}}$$

where RLD represents Root Length Density (cm·cm^−3^), L is the total root length in the sample (cm), and V_s_ is the soil sampling volume (cm^3^; 2000 cm^3^ in this study).

#### 2.5.2 Root surface area density (RSAD)

Root Surface Area Density(RSAD) is defined as the total root surface area per unit volume of soil and is used to characterize the extent of contact between roots and soil. Because water and nutrients are primarily absorbed through the root surface, RSAD is widely applied to evaluate the potential absorptive capacity of root systems and is an important functional structural parameter of roots (Pregitzer et al., 2002; Fitter, 2002). The calculation is shown in Equation (2):


$$\text{RSAD}=\frac{\text{SA}}{{\text{V}}_{\text{s}}}$$

where RSAD represents Root Surface Area Density (cm^2^·cm^−3^), SA is the total root surface area in the sample (cm^2^), and V_s_ is the soil sampling volume (cm^3^; 2000 cm^3^ in this study).

#### 2.5.3 Root volume density (RVD)

Root Volume Density(RVD) refers to the total volume of roots per unit volume of soil and describes the volumetric distribution of roots within the soil profile. This indicator integrates information on root thickness and spatial occupancy and can be used to analyze root structural characteristics and their effects on soil pore structure and water movement processes (Dexter, 1987; Arsenault et al., 1995). The calculation is shown in Equation (3):


$$\text{RVD}=\frac{\text{RV}}{{\text{V}}_{\text{s}}}$$

where RVD represents Root Volume Density (mm^3^·cm^−3^), RV is the total root volume in the sample (mm^3^), and V_s_ is the soil sampling volume (cm^3^; 2000 cm^3^ in this study).

#### 2.5.4 Root dry mass density (RDMD)

Root Dry Mass Density(RDMD) is defined as the dry mass of roots per unit volume of soil and is used to characterize the accumulation of root biomass within the soil. This parameter reflects belowground biomass allocation and is an important indicator for evaluating root growth intensity and its response to environmental conditions (Jackson et al., 1997; Vogt et al., 1998). The calculation is shown in Equation (4):


$$\text{RDMD}=\frac{\text{DM}}{{\text{V}}_{\text{s}}}$$

where RDMD represents Root Dry Mass Density (mg·cm^−3^), DM is the root dry mass in the sample (mg), and V_s_ is the soil sampling volume (cm^3^; 2000 cm^3^ in this study).

### 2.6 Stability score

To comprehensively evaluate the interannual stability of root traits in yield prediction, a stability score (0-100) was developed in this study as a heuristic composite index rather than a formal statistical test. The score integrates four aspects: overall correlation strength, mean annual correlation strength, directional consistency across years, and significance frequency. The calculation is shown in Equation (5):


$$\text{Stability Score}=40\times \left|{\text{r}}_{\text{all}}\right|+30\times \bar{|{\text{r}}_{\text{year}}|}+15\times \text{D}+15\times \frac{{\text{N}}_{\text{sig}}}{{\text{N}}_{\text{year}}}$$

where ${\text{∣r}}_{\text{all}}|$ is the absolute Pearson correlation coefficient between the trait and yield across the full dataset; $\bar{{\text{∣r}}_{\text{year}}|}$ is the mean absolute Pearson correlation coefficient across years; D denotes the directional consistency of correlations across years, which was assigned a value of 1 when the correlation signs were consistent among years and 0 otherwise; *N_sig_* is the number of years with significant correlations, and *N_year_* is the total number of years. Statistical significance was defined as p<0.05.

In this scoring system, the weights assigned to overall correlation strength, mean annual correlation strength, directional consistency, and significance frequency were 40%, 30%, 15%, and 15%, respectively. Considering that the stability ranking may be affected by the assigned weighting scheme, this study further conducted a sensitivity analysis using several alternative weighting schemes, including an equal-weight scheme, a correlation-heavy scheme, an interannual-stability-heavy scheme, and a significance-support-heavy scheme. Trait rankings under different weighting schemes were compared using mean rank, rank standard deviation, best rank, and worst rank to evaluate the robustness of root-trait prioritization based on the stability score.

### 2.7 Model construction and training methods

Before model construction, the nested root observations were aggregated to the plot-year scale, and plot-year observations were used as the basic analytical units for yield prediction modeling. To ensure consistency between the modeling dataset and the experimental design, the aggregated data were structurally checked, confirming that the final dataset contained 48 plot-year observations, corresponding to four years, four treatments, and three plot replicates. Each plot-year observation corresponded to one grain yield observation, while root traits from different soil layers were used as feature variables for that observation unit. This procedure avoided treating multiple sampling points or root-layer records as independent yield-level samples.

To reduce the risk of information leakage caused by random splitting of nested observations, a Leave-One-Year-Out cross-validation strategy grouped by year was used as the main validation approach. Specifically, in each outer validation fold, all plot-year observations from one complete year were held out as the test set, while observations from the remaining years were used as the training set. Within the outer training set, GroupKFold grouped by year was further used for hyperparameter tuning and feature selection. Feature selection was embedded within the model-training workflow and included two options: retaining all candidate features or applying SelectKBest based on univariate f-regression. The number of retained features was treated as one of the tunable parameters (Kuhn and Johnson, 2013). This procedure ensured that information from the held-out test year was not used during feature selection or model tuning.

**Figure 2:**
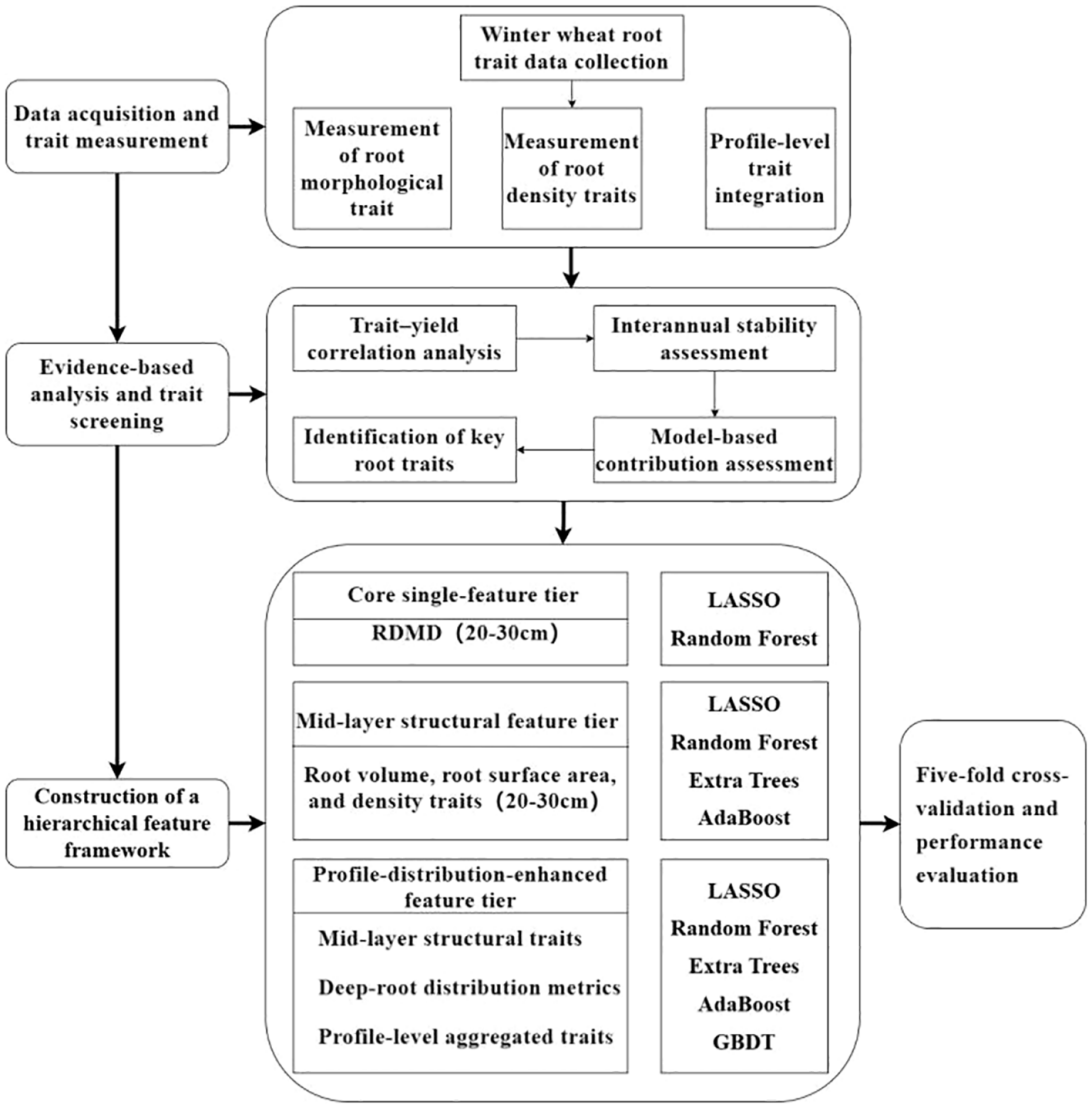
Technical workflow for hierarchical root-trait-based winter wheat yield prediction.

To compare the ability of different model types to characterize the relationship between root traits and grain yield, a candidate model set was constructed, including baseline models, linear models, regularized regression models, robust regression models, kernel-based methods, distance-based models, and ensemble tree models. Specifically, the models included DummyMean, Linear Regression, Ridge, LASSO, Elastic Net, Bayesian Ridge, Huber Regression, Polynomial Ridge, Spline Ridge, Kernel Ridge, SVR, KNN, Random Forest, Extra Trees, Gradient Boosting Decision Trees, HistGradientBoosting, AdaBoost, and XGBoost when available. All models were evaluated under the same grouped cross-validation framework using R^2^, RMSE, and MAE as performance metrics (Kuhn and Johnson, 2013). Fold-wise predictions, mean ± standard deviation, 95% confidence intervals, paired Wilcoxon tests with Holm correction. Model construction and implementation were carried out in a Python environment and developed and executed in PyCharm 2024 (**Figure 2**).

#### 2.7.1 Construction of the hierarchical root-trait feature framework

Based on the results of correlation analysis, interannual stability evaluation, and model contribution analysis, a hierarchical root-trait feature framework was constructed to systematically organize candidate predictors. This framework was developed based on the principles of representativeness, stability, and completeness of profile structural information, and it jointly considered the association strength between root traits and grain yield, the consistency of their interannual performance, and their contributions in multivariate models. It should be noted that these analyses were mainly used for root-trait prioritization and candidate feature framework construction, rather than as a predictor preselection procedure before cross-validation.

During feature organization, root traits showing relatively consistent performance in terms of correlation strength, interannual stability, and model contribution were preferentially included, while redundant inclusion of highly collinear indicators was minimized as far as possible. Meanwhile, root distribution characteristics from different soil layers and profile-level aggregate indicators were incorporated to complement profile structural information that could not be adequately represented by a single soil layer or a single trait. Based on these principles, root-trait predictors were organized into three hierarchical tiers: the core single-feature tier, the mid-layer structural tier, and the profile distribution–enhanced tier, for subsequent model comparison and evaluation under different feature combinations.

#### 2.7.2 Linear sparse regression model: LASSO

To establish a set of interpretable and competitive baseline models, linear sparse regression, regularized linear regression, kernel-based regression, and distance-based regression were included in the modeling framework. The Least Absolute Shrinkage and Selection Operator (LASSO) was used as the primary sparse linear model. LASSO introduces an L1 regularization term into the loss function and can be expressed as:


$$\text{Minimize: J}(\beta )=\sum_{\text{i}=1}^{\text{n}}{\left({\text{y}}_{\text{i}}-{\text{x}}_{i}^{\text{T}}\beta \right)}^{2}+\lambda \sum_{\text{j}=1}^{\text{p}}\left|\beta_{\text{j}}\right|$$

where y is the dependent variable, X represents the independent variables, β denotes the regression coefficients, and λ is the regularization parameter. By simultaneously performing coefficient shrinkage and variable selection, LASSO is able to identify representative predictors in the presence of multicollinearity (Tibshirani, 1996).Given that root traits across different soil layers are often highly correlated, the LASSO model was primarily used to evaluate the linear predictive ability of root traits under simplified feature sets and to assist in identifying redundant features and key root traits contributing to yield prediction (Dormann et al., 2013).

To complement LASSO, Ridge regression and Elastic Net were also introduced as additional regularized linear models. Ridge regression applies L2 regularization and is effective for stabilizing coefficient estimation under multicollinearity, whereas Elastic Net combines L1 and L2 penalties and is therefore more flexible in balancing sparsity and coefficient shrinkage. In the present study, LASSO, Ridge, and Elastic Net were implemented as pipelines combining StandardScaler with LassoCV, RidgeCV, and ElasticNetCV, respectively. For Ridge regression, candidate penalty values were defined on a logarithmic grid from 10–^3^ to 10^3^ (60 values). For Elastic Net, the mixing parameter l1_ratio was searched over [0.1,0.3,0.5,0.7,0.9], while the regularization parameter α was searched over a logarithmic grid from 10–^3^ to 10^2^ (60 values). For LASSO and Elastic Net, the maximum number of iterations was set to 50,000.

In addition, two support vector regression (SVR) models and one K-nearest neighbors (KNN) model were included to represent kernel-based and distance-based prediction approaches. Specifically, an RBF-kernel SVR was implemented with C = 30.0, ϵ=0.1, and gamma = “scale”, whereas a linear-kernel SVR was implemented with C = 10.0 and ϵ=0.1. Both SVR models were fitted after predictor standardization. KNN regression was implemented with k=7 and distance-based weighting. These models were incorporated to provide additional comparisons for both approximately linear and moderately nonlinear trait–yield relationships under a unified reproducible framework.

#### 2.7.3 Bagging-based ensemble tree models: random forest and extra trees

Random Forest (RF) and Extremely Randomized Trees (Extra Trees) were employed as Bagging-based ensemble tree models to capture nonlinear responses and feature interactions between root traits and winter wheat yield (Breiman, 2001). RF constructs an ensemble of decision trees based on bootstrap resampling of the training data and random trait screening at node splits, thereby reducing model variance and improving predictive robustness. Extra Trees follows a similar ensemble strategy but introduces greater randomness by selecting split thresholds more randomly, which may further reduce sensitivity to sample-specific variation and improve robustness in complex feature spaces (Geurts et al., 2006; Breiman, 2001; Jeong et al., 2016; Liakos et al., 2018).

In the current implementation, both RF and Extra Trees were fitted using fixed parameter settings rather than exhaustive grid search. Specifically, n_estimators was set to 1200, min_samples_leaf was set to 2, random_state was fixed at 42, and n_jobs = -1 was used to enable parallel computation. These settings were adopted to provide stable nonlinear benchmarks under the relatively limited sample size of the present study. Therefore, the reported results for RF and Extra Trees should be interpreted as model performance under predefined and reproducible parameter configurations rather than under a full hyperparameter optimization procedure.

#### 2.7.4 Boosting-based ensemble learning models and model evaluation workflow

To further characterize complex nonlinear responses and interaction effects among root traits, two Boosting-based ensemble learning models, Gradient Boosting Decision Trees (GBDT) and AdaBoost, were also included. GBDT constructs an additive predictive model by sequentially fitting new trees to the residuals of previous trees, thereby progressively reducing prediction error and effectively approximating nonlinear relationships (Friedman, 2001). AdaBoost iteratively updates sample weights during training so that observations with larger prediction errors receive greater attention in subsequent rounds, which enhances sensitivity to difficult-to-predict samples (Freund and Schapire, 1997; Chen and Guestrin, 2016).

In this study, GBDT was implemented with n_estimators = 800, learning_rate = 0.03, max_depth = 3, and random_state = 42. AdaBoost was implemented with n_estimators = 600, learning_rate = 0.03, and random_state = 42. As with the Bagging-based models, these parameter values were directly specified in the script and were not selected through exhaustive grid search. Thus, GBDT and AdaBoost served as fixed and reproducible Boosting configurations for comparative analysis.

Before model fitting, all predictor variables were converted to numeric format. Features with insufficient valid observations or zero variance were removed to improve model stability. Specifically, variables with fewer than max(10, 40% of the total sample size) valid observations were excluded, and the remaining missing values were imputed using the mean of each feature. Grain yield was used as the response variable, and the predictor set for each model was defined according to the pre-established root-trait feature tiers. Model evaluation was conducted using shuffled K-fold cross-validation with a fixed random seed (random_state = 42), and the number of folds was determined according to sample size. For each fold, model performance was assessed using the coefficient of determination (R^2^), root mean square error (RMSE), and mean absolute error (MAE). Fold-level results were retained to calculate the mean, standard deviation, and 95% confidence interval of each metric, thereby providing a more complete assessment of model stability than single-value point estimates alone.

In addition, the final fitted parameter settings of each model were exported to facilitate reproducibility. This workflow enabled simultaneous comparison of linear, kernel-based, distance-based, Bagging-based, and Boosting-based models under a unified and reproducible evaluation framework. Among these candidate models, the tree-based ensemble methods showed comparatively stronger performance and were therefore emphasized in the Results section.

#### 2.7.5 Model training and performance evaluation

All models were trained and evaluated using 5-fold cross-validation to ensure robust performance on independent validation subsets and to avoid overfitting caused by limited sample size (Hastie et al., 2009). Model predictive performance was comprehensively assessed using the coefficient of determination (R^2^), root mean square error (RMSE) and mean absolute error (MAE). Let y denote the observed values and ŷ the predicted values; the evaluation metrics were calculated as follows:


$$\text{MAE}=\frac{1}{\text{m}}\sum_{\text{i}=1}^{\text{m}}\left|{\text{y}}_{\text{i}}-\hat{{\text{y}}_{\text{i}}}\right|$$

$$\text{RMSE}=\sqrt{\frac{1}{\text{m}}\sum_{\text{i}=1}^{\text{m}}{\left({\text{y}}_{\text{i}}-\hat{{\text{y}}_{\text{i}}}\right)}^{2}}$$

$${\text{R}}^{2}=1-\frac{\sum_{\text{i}=1}^{\text{m}}{\left({\text{y}}_{\text{i}}-\hat{{\text{y}}_{\text{i}}}\right)}^{2}}{\sum_{i=1}^{m}{\left({\text{y}}_{\text{i}}-\bar{{\text{y}}_{\text{i}}}\right)}^{2}}$$

Lower values of MAE and RMSE indicate better model performance, whereas R^2^ values closer to 1 represent higher predictive accuracy. For each root-trait feature hierarchy, the model exhibiting the best performance under cross-validation was selected as the recommended predictive model for that feature level. This multi-model comparison framework facilitates a systematic evaluation of how increasing root-trait complexity—from a single core trait to a profile-enhanced feature set—affects the accuracy of winter wheat yield prediction.

#### 2.7.6 Interpretability analysis of the best-performing model

To enhance the interpretability of the best-performing model, feature attribution analysis was further conducted for the model with the highest predictive performance. Specifically, SHAP was preferentially used to evaluate the relative contribution of each feature to the model predictions. If SHAP was unavailable, failed to compute, or was not suitable for the current model, Permutation Importance was used as an alternative. Based on this analysis, the mean importance of each feature was calculated and ranked, thereby identifying the key root traits that contributed most strongly to the prediction results of the best-performing model.

## 3 Results

### 3.1 Comprehensive screening of relationships between root traits and winter wheat yield

In this study, a comprehensive descriptive statistical analysis was conducted for winter wheat root traits and grain yield to evaluate the effects of different root traits on yield and their variability characteristics (**Table 2**). Among root density–related indicators, the coefficients of variation (CV) for RLD, RSAD, RVD, and RDMD ranged from 20.69% to 27.07%, indicating moderate levels of variability. Specifically, RVD exhibited the highest CV (27.07%), whereas RDMD showed a relatively low CV (20.69%). Pronounced differences between the minimum and maximum values were observed for all indicators, indicating considerable variability among samples.

**Table 2:** Descriptive statistics of winter wheat root traits and yield.

Raw Name	Mean ± SD	CV (%)	Min	Max
Root length (cm)	24376.33 ± 5827.93	23.91	6253.75	38241.02
Root surface area (cm^2^)	1673.65 ± 399.20	23.85	466.88	3073.13
Root volume (cm^3^)	9.39 ± 2.54	27.07	2.80	20.18
Root dry mass (g)	1.30 ± 0.27	20.69	0.59	1.85
RLD (cm cm^−3^)	2.03 ± 0.49	23.91	0.52	3.19
RSAD (cm^2^ cm^−3^)	0.14 ± 0.03	23.85	0.04	0.26
RVD (mm^3^ cm^−3^)	0.78 ± 0.21	27.07	0.23	1.68
RDMD (mg cm^−3^)	0.11 ± 0.02	20.69	0.05	0.15
Grain yield (kg ha-1)	3588.45 ± 1056.00	29.43	1453.8	4751.1

For absolute root traits, including root length, root surface area, root volume, and root dry mass, wide ranges of variation were also observed. Root length varied from 6,253.75 to 38,241.02 cm, with a CV of 23.91%, while root surface area ranged from 466.88 to 3,073.13 cm^2^, with a CV of 23.85%. The coefficients of variation for root volume and root dry mass were 27.07% and 20.69%, respectively, indicating considerable differences among samples in root system size and biomass accumulation.

Among all variables, winter wheat yield exhibited the largest variability, with a CV of 29.43%. Yield ranged from 1,453.80 to 4,751.10 kg·ha^−^¹, reflecting substantial differences in winter wheat productivity under different years and treatment conditions. Overall, both root traits and yield demonstrated sufficient variability, providing a reliable data basis for subsequent analyses of root trait–yield relationships, interannual stability assessment, and the development of multivariate predictive models. 

**Figure 3:**
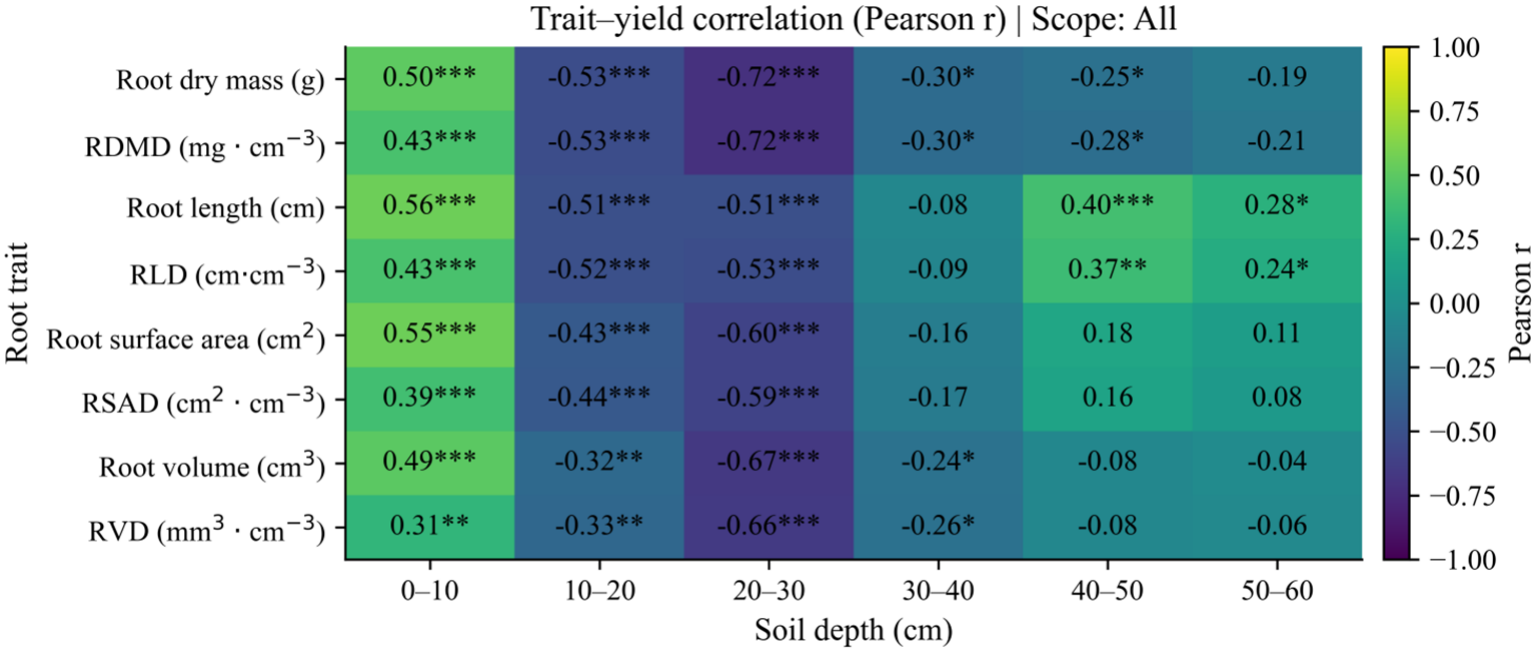
Distribution of correlations between winter wheat root traits and yield across different soil layers. ***p ≤ 0.001, **p ≤ 0.01, *p ≤ 0.05.

Based on the above descriptive statistics, further screening was conducted to identify the root traits most closely associated with winter wheat yield across different soil layers. To comprehensively identify root traits strongly related to yield, correlation analyses were performed for all root indicators across different soil layers (**Figure 3**).

The overall correlation analysis revealed pronounced depth-dependent differences in the relationships between root traits and yield (**Figure 3**). In the 0–10 cm surface soil layer, most root traits showed significant positive correlations with yield. Specifically, root length, root surface area, and root dry mass exhibited correlation coefficients greater than 0.50, all reaching highly significant levels (p< 0.001). RDMD, RLD, and RVD in this layer were also significantly positively correlated with yield, although with slightly lower correlation strengths. In the 10–20 cm and 20–30 cm soil layers, the relationships between root traits and yield generally shifted to significant negative correlations. The negative correlations were most pronounced in the 20–30 cm layer, where the absolute values of correlation coefficients for most traits exceeded 0.60 and all reached highly significant levels (p< 0.001). Root dry mass, root volume, RDMD, and RVD in this layer showed correlation coefficients ranging from −0.66 to −0.72, indicating a highly consistent negative correlation pattern. In the 30–40 cm layer, the overall correlations weakened, with most root traits showing correlation coefficients close to zero; only a few traits reached significance, and their correlation strengths were relatively low. In the 40–50 cm layer, some traits (e.g., root length and RLD) again exhibited significant positive correlations with yield, with correlation coefficients of approximately 0.37–0.40, reaching significant or highly significant levels, whereas the remaining traits showed weak correlations. In the deepest 50–60 cm layer, most root traits had low correlation coefficients with yield and did not reach significance. Overall, the relationships between root traits and yield exhibited clear spatial differentiation along the soil profile: significant positive correlations predominated in the surface layer (0–10 cm), significant negative correlations dominated in the middle layers (10–30 cm), and correlations generally weakened and became more differentiated in deeper soil layers. These results indicate pronounced layer-specific differences in the contribution of root traits to the yield-related correlation pattern.

### 3.2 Interannual stability analysis of root trait–yield relationships

**Figure 4:**
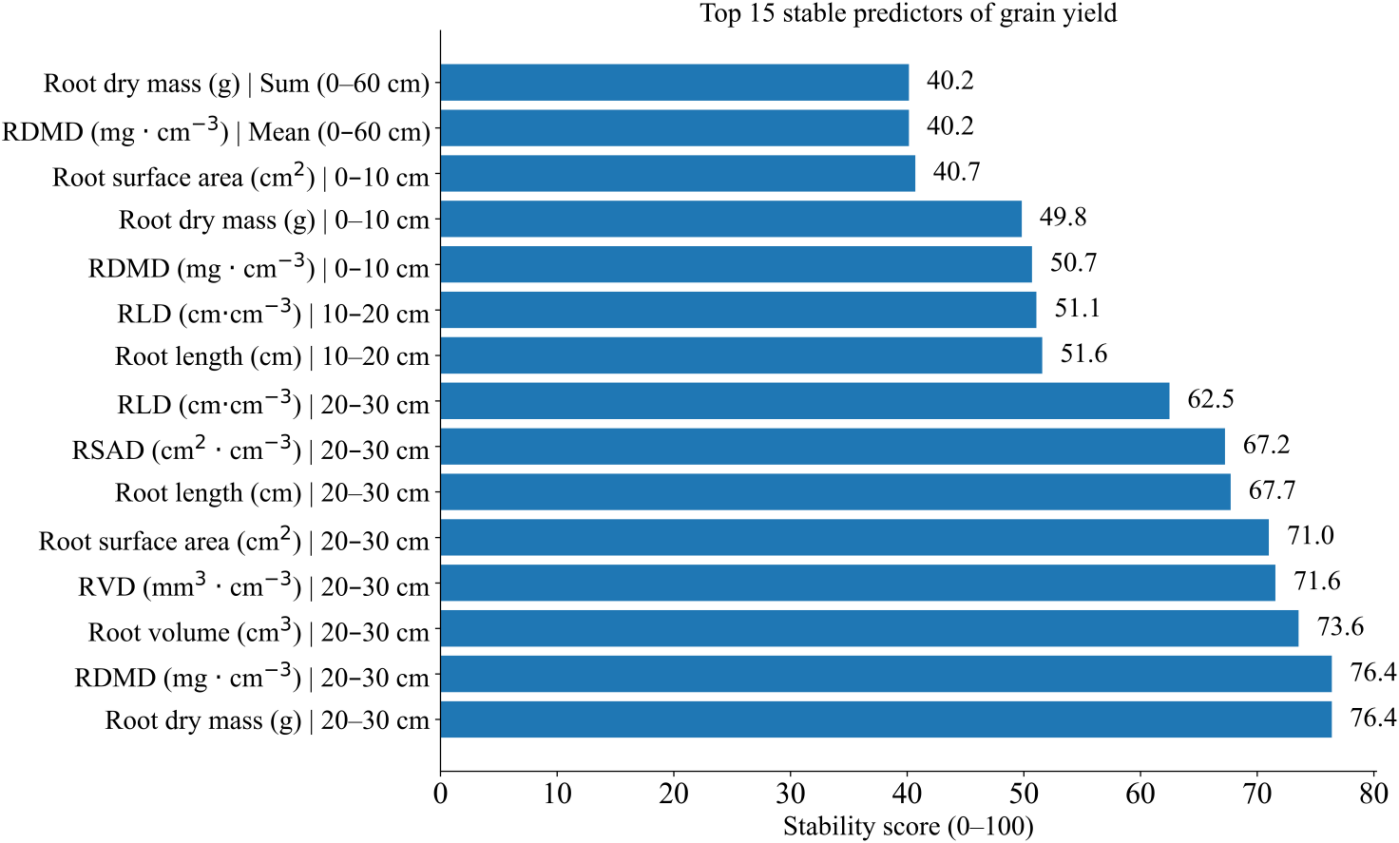
Stability ranking of key root traits in winter wheat yield prediction.

The stability analysis revealed clear soil-depth-dependent differences in the interannual stability of the relationships between root traits and grain yield (**Figure 4**). Among the top 15 indicators ranked by stability score, root traits from the 20–30 cm soil layer were dominant and occurred most frequently in the high-stability score range. Specifically, several root traits from the 20–30 cm layer, including root dry mass, RDMD, root volume, RVD, root surface area, and root length, ranked among the top positions, indicating that root morphological and biomass-related traits in this layer had relatively strong and stable associations with grain yield.

In contrast, although some root traits from the 0–10 cm and 10–20 cm layers also appeared among the top 15 indicators, their overall stability scores were relatively lower. For example, root dry mass, root surface area, and RDMD in the 0–10 cm layer showed moderate stability, while root length and RLD in the 10–20 cm layer exhibited slightly higher stability but still remained below the corresponding indicators from the 20–30 cm layer. In addition, several profile-scale indicators, such as total root dry mass and mean RDMD across the 0–60 cm profile, were also included among the top-ranked stable traits. However, their stability scores were generally lower than those of the stratified root traits from the 20–30 cm layer, suggesting that simple whole-profile aggregate indicators did not show stronger interannual stability.

Further weighting sensitivity analysis showed that the above stability ranking was relatively robust to the choice of weighting scheme (**Table 3**). The sensitivity analysis of the stability score showed that the ranking of major root traits remained generally stable across different weighting schemes. The top eight traits were all derived from the 20–30 cm soil layer, indicating that the dominance of root traits in this layer was not driven by a single weighting scheme. Among them, RDMD and root dry mass at 20–30 cm consistently ranked as the top two traits, with MeanRank values of 1.2 and 1.8, respectively, and both had a MeanScore of 88.67, suggesting that they formed the most stable core root-trait combination. The SDRank values of RVD, RootVolume, RSAD, and SurfArea at 20–30 cm were all 0, indicating that their rankings were completely consistent across different weighting schemes and showed strong ranking robustness.

**Table 3:** Ensitivity analysis of root-trait stability ranking under alternative weighting schemes.

Feature	Meanrank	SDrank	BestRank	Worstrank	Meanscore
RDMD:20–30	1.2	0.45	1	2	88.67
Mass:20–30	1.8	0.45	1	2	88.67
RVD:20–30	3.0	0.00	3	3	81.94
RootVolume:20–30	4.0	0.00	4	4	81.54
RSAD:20–30	5.0	0.00	5	5	77.00
SurfArea:20–30	6.0	0.00	6	6	76.51
RLD:20–30	7.2	0.45	7	8	66.58
Length:20–30	7.8	0.45	7	8	66.22
RDMD:0–10	9.0	0.00	9	9	64.34
RLD:10–20	10.0	0.00	10	10	62.47

MeanRank indicates the average rank under different weighting schemes, with smaller values representing higher rankings. SDRank indicates the standard deviation of ranks, with smaller values indicating greater ranking stability. BestRank and WorstRank represent the best and worst ranks under different weighting schemes, respectively. MeanScore and SDScore represent the mean and standard deviation of the stability scores under different weighting schemes, respectively. The data were derived from the sensitivity analysis results.

Overall, the stability score and its sensitivity analysis jointly indicated that the 20–30 cm soil layer represented the key root-zone interval with the most pronounced stable association between root traits and grain yield under the present experimental conditions. Surface-layer root traits and profile-scale aggregate indicators tended to act more as secondary stability factors. These results support prioritizing root traits in the 20–30 cm layer in subsequent root-trait screening and yield-association analyses.

### 3.3 Model-based contribution analysis of key root traits

**Figure 5:**
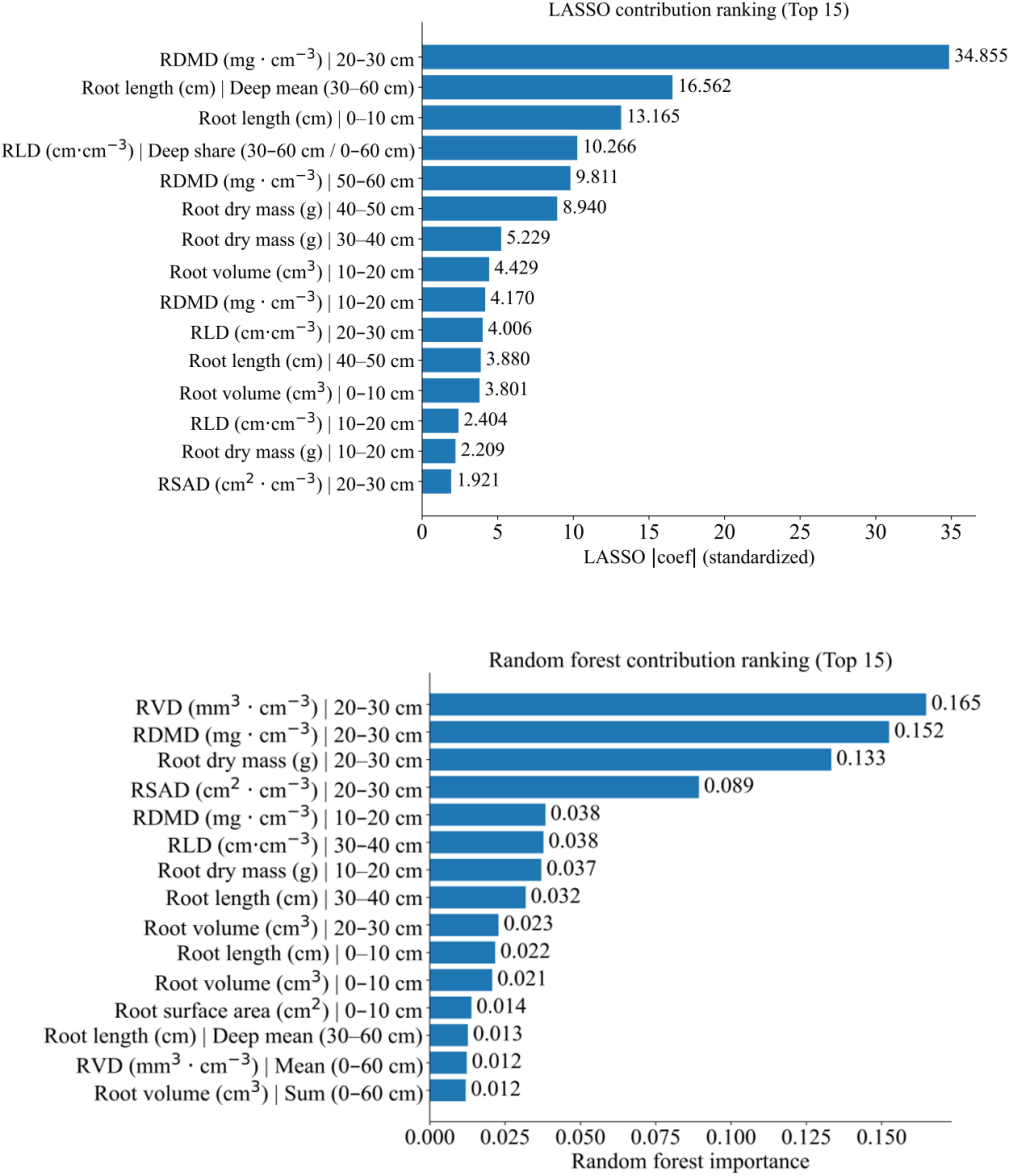
Comparison of key root traits for winter wheat yield identified by LASSO and random forest models.

After clarifying the relationships between individual root traits and winter wheat yield and their interannual stability, multivariate modeling approaches were further employed to comprehensively evaluate the relative contributions of different root traits to yield prediction (**Figure 5**), with the aim of identifying key root characteristics that play dominant roles in yield formation.

Overall, results from the multivariate models indicated significant differences in the contributions of individual root traits to winter wheat yield prediction, while consistently exhibiting a clear layer-dependent pattern. Based on the outputs of both the LASSO and Random Forest models, the contributions of root traits to yield varied markedly among soil layers. Both models consistently identified root traits from the 20–30 cm soil layer as dominant in importance rankings. Notably, RDMD in the 20–30 cm layer showed the highest absolute standardized regression coefficient in the LASSO model and ranked among the most important features in the Random Forest model, demonstrating strong consistency across modeling approaches.

According to the variable selection results of the LASSO model, the top 15 predictors were mainly concentrated in mid-layer (20–30 cm) and surface-layer (0–10 cm) root traits, while also including several profile-scale indicators, such as mean deep root length and deep-layer proportion metrics for the 30–60 cm profile. Overall, variables selected by LASSO were predominantly quantitative traits, including RDMD, root length, and root volume, reflecting relatively stable linear relationships between yield and root traits. In contrast, the feature importance ranking from the Random Forest model revealed greater diversity in the types of variables identified as important. In addition to root dry mass and RDMD, RVD and RSAD from the 20–30 cm layer also exhibited high importance values. Moreover, several profile-scale indicators (e.g., mean or total values across the 0–60 cm profile) appeared in the Random Forest rankings, indicating that this model is more inclusive of information across different spatial scales during variable selection.

Taken together, results from both models consistently identified the 20–30 cm soil layer as a key depth across different modeling approaches, whereas surface and deep root traits exhibited complementary patterns depending on the model used. LASSO tended to emphasize root traits with significant linear effects, while Random Forest highlighted the relative importance of multiple trait types in overall prediction performance. Nevertheless, both models showed high consistency in identifying the key soil layer. It should be noted that the standardized regression coefficients from LASSO and the feature importance measures from Random Forest have different statistical interpretations; therefore, their numerical values were not directly compared in this study. Instead, their respective ranking results were presented. By jointly examining outputs from both models, key root traits and their associated soil layer distributions that consistently emerged across different modeling frameworks could be more comprehensively identified.

### 3.4 Root trait–based feature framework and yield prediction performance

#### 3.4.1 Construction of hierarchical root-trait feature tiers

By integrating the results of correlation analysis, interannual stability evaluation, weighting sensitivity analysis, and model-based contribution analysis, several root traits with relatively stable associations with winter wheat grain yield were identified and used as the basis for constructing hierarchical feature tiers. Overall, root traits with higher stability and model contribution were mainly concentrated in the 20–30 cm soil layer. Among them, RDMD, root dry mass, root volume, RVD, and root surface area at 20–30 cm showed consistently high relevance across different analytical frameworks. In particular, RDMD at 20–30 cm exhibited a strong association with grain yield, good interannual consistency, and relatively high model-based contribution, suggesting that it could serve as a core candidate trait for characterizing root–yield relationships under the present experimental conditions. In addition, some deeper-layer traits and profile-level aggregate indicators also showed certain contributions, indicating that root distribution information from deeper soil layers may provide complementary structural information, although their stability and ranking robustness were generally weaker than those of the 20–30 cm traits.

Based on these findings, root-trait predictors were organized into three hierarchical feature tiers to support subsequent model comparison under different levels of data availability and model complexity (**Table 4**). The first tier was the core single-feature tier, represented by RDMD at 20–30 cm, and was used to evaluate whether a single representative root indicator could capture yield-related variation. The second tier was the mid-layer structural tier, which integrated multiple representative root traits from the 20–30 cm soil layer, including biomass-, morphology-, and density-related indicators, to characterize the key root-zone structure. The third tier was the profile distribution–enhanced tier, which further incorporated deep-layer mean traits, profile-level aggregate indicators, and deep-layer proportion indicators based on the mid-layer structural traits. These tiers were not mutually exclusive, but represented successive expansions of root-trait information from a single core indicator to a more complete profile-based representation. 

**Table 4:** Hierarchical root trait feature combinations for different yield prediction objectives.

Feature level	Main feature components	Data requirement intensity	Model complexity	Applicable prediction scenarios
Core single-feature layer(Tier1)	RDMD (20-30cm)	Low	Low	Rapid assessment; limited sample size; constrained field investigations
Mid-layer structural feature layer(Tier2)	Root volume, root surface area, and corresponding density traits in the 20–30 cm layer (8 variables)	Medium	Medium	Conventional yield prediction; structural analysis
Profile distribution–enhanced feature layer(Tier3)	Mid-layer structural traits + deep root distribution indicators + profile-level aggregated features	High	High	High-precision prediction; machine learning modeling; multi-year generalization

#### 3.4.2 Predictive performance across hierarchical feature tiers

Under the year-grouped Leave-One-Year-Out cross-validation framework, predictive performance differed among the hierarchical root-trait feature tiers (**Table 5**). Overall, as feature information was progressively expanded from the core single-feature tier to the mid-layer structural tier and then to the profile distribution–enhanced tier, the overall predictive performance of the models improved. This indicates that a single key root trait could provide certain yield-related information, while integrating multidimensional root structural traits from the key 20–30 cm soil layer and further incorporating deep-layer root distribution information helped improve the ability of the models to explain variation in winter wheat yield.

**Table 5:** Comparison of the best-performing machine learning models under different feature hierarchies.

Tier	Model	Feature number	R^2^ mean ± SD	R^2^ 95% CI	RMSE mean ± SD (kg ha^−^¹)	RMSE 95% CI	MAE mean ± SD (kg ha^−^¹)	MAE 95% CI
Tier1	ExtraTrees	1	0.69 ± 0.31	0.19–1.19	13.57 ± 3.85	7.45–19.70	10.72 ± 2.66	6.49–14.95
Tier1	GBDT	1	0.61 ± 0.37	0.03–1.19	17.23 ± 8.08	4.38–30.08	11.66 ± 4.13	5.08–18.24
Tier1	KNN	1	0.60 ± 0.53	−0.244–1.43	14.70 ± 5.59	5.80–23.60	11.99 ± 4.97	4.09–19.90
Tier2	AdaBoost	8	0.73 ± 0.28	0.28–1.18	11.77 ± 1.13	9.98–13.57	9.76 ± 1.32	7.66–11.85
Tier2	ExtraTrees	8	0.70 ± 0.31	0.21–1.19	13.41 ± 4.06	6.95–19.87	10.60 ± 2.86	6.04–15.15
Tier2	GBDT	8	0.65 ± 0.33	0.12–1.18	12.60 ± 2.01	9.40–15.81	9.72 ± 0.94	8.22–11.22
Tier3	AdaBoost	18	0.74 ± 0.28	0.29–1.19	11.51 ± 1.56	9.03–13.99	9.32 ± 1.89	6.30–12.33
Tier3	GBDT	18	0.73 ± 0.27	0.31–1.16	11.53 ± 0.43	10.84–12.21	8.99 ± 0.53	8.16–9.83
Tier3	ExtraTrees	18	0.69 ± 0.31	0.19–1.19	13.57 ± 3.85	7.45–19.70	10.72 ± 2.66	6.49–14.95
Tier3	XGBoost	18	0.64 ± 0.44	−0.06–1.33	13.11 ± 2.52	9.11–17.12	10.46 ± 1.80	7.60–13.33
Tier3	KNN	18	0.63 ± 0.55	−0.25–1.51	11.78 ± 3.84	5.68–17.88	9.54 ± 2.93	4.87–14.21

In the core single-feature tier (Tier1), RDMD at 20–30 cm was used as the only predictor. In this tier, ExtraTrees showed the best performance, with an R^2^ of 0.69 ± 0.31, an RMSE of 13.57 ± 3.85 kg·ha^-1^, and an MAE of 10.72 ± 2.66 kg·ha^-1^. GBDT and KNN also showed certain predictive ability, with R^2^ values of 0.61 ± 0.37 and 0.60 ± 0.53, respectively. These results indicate that RDMD at 20–30 cm alone could capture part of the root-related information associated with yield. However, the relatively large standard deviations and confidence intervals of R^2^ in this tier suggest that the predictive stability of a single root trait across years remained limited and was insufficient to fully represent the complex variation in winter wheat yield formation.

Compared with Tier1, the mid-layer structural feature tier (Tier2), which integrated eight representative root traits from the 20–30 cm soil layer, improved model performance. In this tier, AdaBoost achieved the best performance, with an R^2^ of 0.73 ± 0.28, an RMSE of 11.77 ± 1.13 kg·ha^-1^, and an MAE of 9.76 ± 1.32 kg·ha^-1^. ExtraTrees and GBDT also performed well, with R^2^ values of 0.70 ± 0.31 and 0.65 ± 0.33, respectively. Compared with Tier1, the overall RMSE and MAE values in Tier2 were reduced, indicating that the combination of multiple morphological, biomass-related, and density-related root traits from the 20–30 cm layer provided a more complete representation of yield-related root structural information than RDMD alone. This result further supports the role of the 20–30 cm soil layer as a key root-zone interval for winter wheat yield prediction.

The profile distribution–enhanced feature tier (Tier3), which further incorporated deep-layer mean traits, profile-level aggregate indicators, and deep-layer proportion indicators based on Tier2, achieved the best overall predictive performance. In this tier, AdaBoost showed the highest R^2^, reaching 0.74 ± 0.28, with an RMSE of 11.51 ± 1.56 kg·ha^-1^ and an MAE of 9.32 ± 1.89 kg·ha^-1^. GBDT showed a very similar performance, with an R^2^ of 0.73 ± 0.27, and achieved the lowest MAE of 8.99 ± 0.53 kg ha^-1^, together with relatively low RMSE variability, indicating more stable prediction errors across years. ExtraTrees, XGBoost, and KNN also maintained moderate to good predictive performance in Tier3, with R^2^ values of 0.69, 0.64, and 0.63, respectively.

Comparisons among the feature tiers showed that the best model performance in Tier3 was slightly higher than that in Tier2. For example, the R^2^ of AdaBoost increased from 0.73 in Tier2 to 0.74 in Tier3, while RMSE decreased from 11.77 to 11.51 kg·ha^-1^ and MAE decreased from 9.76 to 9.32 kg·ha^-1^. Although the magnitude of improvement was relatively small, these results suggest that deep-layer root distribution and profile-level aggregate indicators provided additional information beyond the 20–30 cm mid-layer root structural traits. Overall, root traits from the 20–30 cm soil layer remained the core information source for yield prediction, whereas deeper-layer and profile-scale indicators mainly played a complementary role in model refinement. Therefore, Tier2 may provide a more cost-effective feature combination when data acquisition is constrained or simplified prediction is required, whereas Tier3 may be more advantageous when more complete root-profile data are available and higher predictive accuracy is desired.

#### 3.4.3 Best-performing model and feature contribution

For the best-performing model, the final interpretability analysis was conducted using the profile distribution–enhanced feature tier combined with AdaBoost. Although this feature tier initially included 18 candidate predictors, the final tuned model retained five features through SelectKBest feature selection. The selected features were all derived from the 20–30 cm soil layer, including RSAD, RVD, RDMD, root volume, and root dry mass. The final tuned parameters were learning_rate = 0.3, loss = square, n_estimators = 100, and SelectKBest with k = 5.

**Figure 6:**
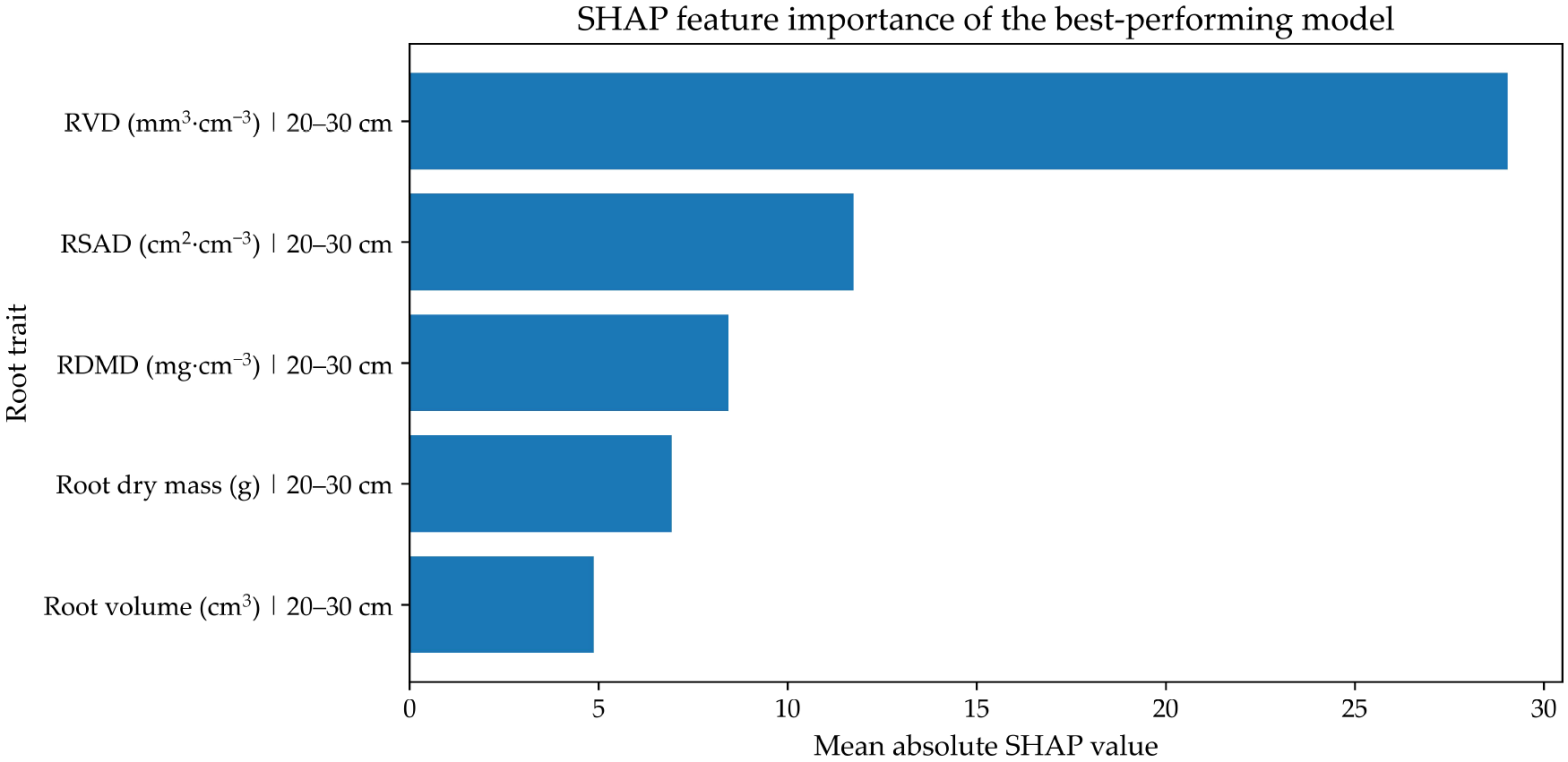
SHAP_feature_importance.

The SHAP-based feature attribution analysis showed that (**Figure 6**) RVD at 20–30 cm had the largest contribution to model prediction, followed by RSAD, RDMD, root dry mass, and root volume at the same soil layer. The mean absolute SHAP value of RVD at 20–30 cm was substantially higher than that of the other selected predictors, indicating that this trait made the strongest average contribution to the prediction output of the AdaBoost model. Similar patterns were observed in the permutation importance analysis, in which RVD and RSAD at 20–30 cm ranked as the two most influential predictors.

These results indicate that, although the profile distribution–enhanced feature tier provided the best cross-validated predictive performance, the final selected predictors were mainly concentrated in the 20–30 cm soil layer. This finding further supports the central role of mid-layer root structural traits in winter wheat yield prediction. The contribution of RVD and RSAD suggests that root volume density and root surface area density in the 20–30 cm layer may provide important structural information for explaining yield variation. It should be noted that feature attribution reflects the contribution of predictors to model output and does not necessarily indicate a positive causal effect on grain yield.

## 4 Discussion

### 4.1 Biological mechanisms underlying the dominance of mid-layer (20–30 cm) root traits in winter wheat yield prediction

This study identified clear soil-depth-dependent differences in the relationships between root traits and winter wheat grain yield. Across correlation analysis, interannual stability evaluation, weighting sensitivity analysis, model-based contribution analysis, and feature-attribution results, root traits from the 20–30 cm soil layer repeatedly showed stronger and more stable yield-related signals than traits from other soil layers. In particular, RDMD, root dry mass, root volume, RVD, RSAD, and root surface area in the 20–30 cm layer were consistently identified as important traits across different analytical procedures. These results suggest that the 20–30 cm soil layer may represent a key functional root-zone interval for winter wheat under the present rainfed field conditions.

It should be emphasized that the importance of a predictive variable does not necessarily indicate a positive effect on yield. Rather, it reflects the strength and stability of the statistical relationship between a given trait and yield variation. In this study, RDMD at 20–30 cm showed a strong and stable association with grain yield across years and modeling approaches, indicating that this trait captured substantial yield-related information. Similar observations have been reported in previous studies showing that root system characteristics can serve as informative indicators of crop productivity and yield variation (Wasson et al., 2012; Comas et al., 2013).

The dominance of 20–30 cm root traits is also consistent with the concept of functional zonation in crop root systems (Gregory et al., 2009). Surface soil layers are strongly affected by precipitation events and evaporative losses, resulting in large temporal fluctuations in water and nutrient availability. Consequently, the contribution of surface roots to yield formation may be unstable under rainfed conditions (Watt et al., 2005). In contrast, although deeper soil layers can buffer water supply during later growth stages, they are not always the primary zone for nutrient acquisition in winter wheat (Kirkegaard et al., 2007). Previous studies have indicated that intermediate soil layers may provide a relatively stable zone for water and nutrient uptake and concentrated root activity (Zhang et al., 2004; Lynch, 2013; Watt et al., 2006). Therefore, the repeated identification of 20–30 cm root traits in this study may reflect the combined effects of root distribution, resource availability, and yield formation processes.

Compared with geometric traits such as root length or root surface area, root dry matter density more directly reflects biomass investment and carbon allocation within a specific soil layer. It may therefore integrate the trade-off between root construction costs and resource acquisition efficiency (Poorter et al., 2012). In some cases, greater root biomass investment in structural tissues may reduce carbon allocation to aboveground growth and grain production (Richards and Passioura, 1989). Such trade-offs between belowground investment and reproductive growth have been widely discussed in relation to plant carbon allocation strategies and the root economics spectrum framework (Poorter et al., 2012; Comas et al., 2013). Therefore, the observed association between RDMD at 20–30 cm and yield should be interpreted as a stable predictive relationship rather than as evidence of a direct positive causal effect.

The SHAP and permutation importance analyses further supported the central role of 20–30 cm root structural traits. For the best-performing AdaBoost model under the profile distribution–enhanced feature tier, the final selected predictors were all derived from the 20–30 cm layer, including RSAD, RVD, RDMD, root volume, and root dry mass. Among these traits, RVD and RSAD showed the largest feature-attribution values, indicating that root volume density and root surface area density in the 20–30 cm layer contributed strongly to model prediction. These findings further support the importance of mid-layer root structural traits in explaining yield variation under the present experimental conditions.

### 4.2 Supporting role of deep root distribution traits in yield prediction

Although 20–30 cm root traits played the dominant role, the profile distribution–enhanced feature tier showed slightly better prediction performance than the mid-layer structural tier. This suggests that deep-layer and profile-level root indicators provided additional information beyond the key mid-layer traits. In the hierarchical feature framework, the inclusion of deep-layer mean traits, profile-level aggregate indicators, and deep-layer proportion indicators improved the representation of root distribution across the soil profile.

Deep roots may not directly determine final yield levels, but they can contribute to maintaining water supply during later growth stages and alleviating drought stress under years with limited precipitation or high environmental variability (Lopes and Reynolds, 2010; Manschadi et al., 2006; Palta et al., 2011). Previous studies have emphasized that deeper roots can improve access to subsoil water and contribute to yield stability under water-limited conditions (Comas et al., 2013). In the present study, deep-layer root traits did not always show the strongest univariate correlations with yield, but their inclusion improved model performance in the profile-enhanced feature tier. This indicates that deep-root information may be more useful when interpreted in combination with mid-layer root traits rather than as isolated predictors.

This result also explains why whole-profile or deep-layer indicators may contribute to multivariate prediction even when their individual correlations with yield are not the highest. Root systems function as integrated structures, and yield formation may depend not only on the magnitude of root traits in a single layer but also on the coordination of root distribution across soil depths. Therefore, deep-layer traits in this study should be interpreted as complementary predictors that refine the structural representation of the root profile. Given the single-site and limited-sample nature of the experiment, however, the supportive role of deep-root traits should be considered preliminary and requires further validation using multi-site and independent datasets.

### 4.3 Implications of differential model responses to root trait information

The framework evaluated different feature tiers using year-grouped Leave-One-Year-Out cross-validation. This strategy reduced the risk of information leakage by ensuring that all observations from one year were held out as the test set in each outer fold. Under this evaluation framework, the prediction results showed a clear trend across the hierarchical feature tiers. The core single-feature tier provided a simple but limited representation of yield-related root information. The mid-layer structural tier improved prediction by combining multiple representative traits from the 20–30 cm layer. The profile distribution–enhanced tier achieved the highest overall performance by incorporating additional deep-layer and profile-level indicators.

The model comparison also showed that tree-based ensemble models and boosting models generally performed better than linear models under the present feature structure. This pattern is broadly consistent with previous studies reporting the usefulness of machine learning methods for crop yield prediction (Jeong et al., 2016). Linear models such as LASSO are useful for identifying sparse and interpretable associations, but their performance can be constrained by multicollinearity among root traits and by nonlinear response patterns that are not explicitly modeled (Liakos et al., 2018). In contrast, tree-based models can more flexibly capture nonlinear relationships and interactions among root traits, while boosting models can progressively improve prediction by learning from weak signals and difficult samples (Shahhosseini et al., 2020).

Among all model–feature tier combinations, AdaBoost under the profile distribution–enhanced feature tier achieved the best cross-validated performance. However, the improvement from the mid-layer structural tier to the profile distribution–enhanced tier was relatively modest. This indicates that 20–30 cm root traits remained the core source of predictive information, whereas deeper-layer and profile-level traits mainly provided complementary refinement. Therefore, the profile-enhanced tier may be useful when complete root-profile measurements are available, whereas the mid-layer structural tier may provide a more practical balance between predictive performance and sampling effort.

It should be noted that model-based feature importance and SHAP-based feature attribution reflect the contribution of predictors to model output, not necessarily the direction or causality of their effects on yield. The high importance of RVD, RSAD, RDMD, root volume, and root dry mass at 20–30 cm therefore indicates that these traits contributed strongly to explaining yield variation within the predictive model. These results should be interpreted as evidence for yield-related predictive relevance rather than as direct proof of causal yield enhancement.

### 4.4 Root trait–based yield prediction strategies for different application scenarios

Based on the integrated analysis of root functional zonation, root trait–yield relationships, interannual stability evaluation, weighting sensitivity analysis, model responses, and feature-attribution results, this study proposed a hierarchical root-trait feature framework for winter wheat yield prediction. By adopting a tiered feature-combination strategy, the framework provides a structured approach for organizing root-trait predictors under different prediction objectives, data availability conditions, and model complexity levels. Under the present experimental conditions, this framework helped reduce the dimensionality of root-trait measurements while maintaining physiological interpretability and reasonable predictive performance.

From an application-oriented perspective, the three feature tiers correspond to different levels of data requirement and analytical complexity. The core single-feature tier, represented by RDMD at 20–30 cm, provides a simplified option for preliminary assessment when root data are limited or field sampling capacity is constrained. This low-input strategy may be suitable for exploratory studies or small-scale experiments where complete root-profile measurement is not feasible. The mid-layer structural tier integrates multiple representative traits from the key 20–30 cm soil layer, including biomass-, morphology-, and density-related indicators. This tier provides a compromise between predictive performance and sampling effort and may therefore be suitable for conventional plot-scale experiments aiming to evaluate root–yield relationships under similar conditions (Van Ittersum et al., 2013).

When more complete root-profile measurements are available, the profile distribution–enhanced tier may further improve the representation of yield variability by integrating deep-layer mean traits, profile-level aggregate indicators, and deep-layer proportion indicators. In the present study, this tier achieved the highest prediction accuracy, suggesting that deep-layer and profile-level root information can provide complementary information beyond mid-layer root traits. However, because the performance improvement over the mid-layer structural tier was relatively modest, the additional sampling effort required for deep-layer and whole-profile measurements should be carefully considered. In practical applications, the choice of feature tier should depend on the research objective, available root data, acceptable sampling effort, and required prediction accuracy.Nevertheless, the framework proposed here should be regarded as a preliminary analytical basis for organizing and prioritizing root traits in root–yield studies, rather than a fixed or universally applicable predictive solution (Van Ittersum et al., 2013). Its broader applicability under different ecological zones, wheat genotypes, soil conditions, and management practices still requires further validation using multi-site experiments and independent datasets. Overall, the main value of this framework lies in identifying a limited number of representative root traits with relatively stable predictive relevance, thereby supporting interpretable and scalable root-trait-based yield analysis under appropriate experimental conditions.

### 4.5 Limitations and future perspectives

Despite the improved modeling workflow and grouped validation strategy, this study has several limitations. First, the experiment was conducted at a single site over four years with four management treatments under rainfed winter wheat conditions. Although the final modeling dataset was aggregated to 48 plot-year observations to match the scale of yield measurements, the sample size remained limited. Therefore, the proposed framework should be interpreted as a site-specific and preliminary predictive approach rather than as a broadly generalizable model. Further validation across different ecological regions, soil types, management practices, years, and genotypes is needed to evaluate the robustness of the identified key root traits and model responses.

Second, root traits were measured at the flowering stage. This stage was selected because winter wheat roots are relatively well developed and closely linked to reproductive growth and yield formation. However, root systems are dynamic throughout the growth cycle, and root traits at other stages, such as jointing and grain filling, may also influence yield formation. Due to the destructive nature of root sampling and the need to maintain consistent sampling across multiple years, this study did not include repeated root measurements across developmental stages. Future studies should examine temporal changes in root traits to better capture the dynamic relationship between root development and yield.

Third, meteorological variables and soil moisture indicators were not explicitly included in the present predictive models. This design was intended to focus on the relative contribution of root traits to yield prediction while reducing the influence of environmental collinearity. Nevertheless, yield formation is jointly influenced by root traits, soil water availability, nutrient supply, climatic conditions, and management practices. Future work should integrate root traits with meteorological data, soil moisture information, soil properties, and remote sensing observations through multi-source data fusion. Recent advances in proximal sensing, remote sensing, and high-throughput phenotyping may provide opportunities to indirectly estimate root-related indicators and expand the applicability of root-trait-based yield prediction (Paez-Garcia et al., 2015; Watt et al., 2020).​

Fourth, although the revised analysis used year-grouped Leave-One-Year-Out cross-validation, fold-wise performance statistics, confidence intervals, and model comparisons, the limited number of year groups constrains the strength of statistical inference. Repeated grouped validation, external validation datasets, and independent multi-site experiments should be adopted in future studies to further evaluate model robustness. In addition, although weighting sensitivity analysis was conducted for the stability score, future work should continue to examine alternative weighting schemes and evaluate whether trait rankings remain stable under broader environmental and management conditions.

In summary, the stratified root-trait framework proposed in this study provides a preliminary basis for combining physiologically meaningful root indicators with predictive modeling. The results highlight the central role of 20–30 cm root structural traits in winter wheat yield prediction under the present experimental conditions, while also indicating that deep-layer and profile-level traits can provide complementary information. Future studies integrating multi-site root observations, environmental variables, and independent validation datasets will be essential for improving the generalizability and practical applicability of this framework.

## 5 Conclusion

Based on multi-layer root trait data aggregated at the plot-year scale, this study examined the relationships between winter wheat root traits and grain yield and developed a hierarchical root-trait feature framework for yield prediction. The results showed clear soil-depth dependency, with root traits in the 20–30 cm layer exhibiting the strongest and most stable associations with yield variation under the present rainfed field conditions. RDMD at 20–30 cm was identified as the most representative single predictive trait, while RVD, RSAD, root dry mass, and root volume in the same layer also showed important model-based contributions. Incorporating multiple mid-layer structural traits improved prediction compared with the single-feature tier, whereas deep-layer and profile-level indicators provided additional but relatively modest complementary information. Under year-grouped Leave-One-Year-Out validation, tree-based ensemble and Boosting models generally outperformed linear models, with AdaBoost under the profile distribution–enhanced tier achieving the best cross-validated performance. Overall, the proposed framework provides a physiologically interpretable and preliminary trait-based reference for organizing root predictors in winter wheat yield modeling under similar experimental conditions. Future studies should further validate this framework across different sites, years, genotypes, and management conditions, and integrate environmental and soil moisture information to improve its robustness and broader applicability.

## Data Availability

The original contributions presented in the study are included in the article/supplementary material. Further inquiries can be directed to the corresponding authors.
